# Co-immunization with two recombinant *Eimeria tenella* lines expressing immunoprotective antigens of *E. maxima* elicits enhanced protection against *E. maxima* infection

**DOI:** 10.1186/s13071-019-3605-6

**Published:** 2019-07-12

**Authors:** Xinming Tang, Chaoyue Wang, Lin Liang, Dandan Hu, Sixin Zhang, Chunhui Duan, Jingxia Suo, Xianyong Liu, Xun Suo, Shangjin Cui

**Affiliations:** 1 Institute of Animal Science, Chinese Academy of Agricultural Sciences, Beijing, China; 2 Key Laboratory of Zoonosis of Ministry of Agriculture & National Animal Protozoa Laboratory, College of Veterinary Medicine, China Agricultural University, Beijing, China; 3 Beijing Scientific Observation and Experimental Station of Veterinary Drugs and Diagnostic Technology, Ministry of Agriculture, Beijing, China

**Keywords:** Recombinant *Eimeria*, Vaccine vector, Apical membrane antigen 1, Immune mapped protein 1, Immune responses

## Abstract

**Background:**

Live anticoccidial vaccines have been a tremendous success for disease prevention. The establishment of the reverse genetic manipulation platform has enabled the development of *Eimeria* parasites, the live anticoccidial vaccine strains, as vaccine vectors. In our previous study, recombinant *E. tenella* expressing a single immunodominant antigen of *E. maxima* (Et-EmIMP1) was able to protect chickens against challenge infection with *E. maxima*. This promising result encouraged us to further explore strategies to improve the protection efficacy of recombinant *Eimeria* and develop it as a vaccine vector.

**Results:**

We constructed a novel recombinant *Eimeria* line expressing apical membrane antigen 1 of *E. maxima* (Et-EmAMA1) and then immunized chickens with Et-EmAMA1 and/or Et-EmIMP1. We found that the *E. maxima* soluble antigen-specific cell-mediated immunity was much stronger in the birds that were co-immunized with Et-EmAMA1 and Et-EmIMP1 than in those that were immunized with Et-EmAMA1 or Et-EmIMP1 alone. The oocyst production after *E. maxima* infection was significantly reduced in the recombinant *Eimeria*-immunized birds compared with the wild-type-immunized and naïve birds. The oocyst production in the birds co-immunized with Et-EmAMA1 and Et-EmIMP1 was consistently the lowest among the treatment groups after *E. maxima* infection.

**Conclusions:**

These results demonstrated that *Eimeria* is an effective vaccine vector that can carry and deliver heterologous *Eimeria* antigens to the host immune system and trigger specific immune responses. Our results also suggested that increasing the number of recombinant *Eimeria* lines is an effective approach to enhance protective immunity against infections with heterologous pathogens.

## Background

The genus *Eimeria* causes coccidiosis in a wide range of domestic and wild animals [1–5]. *Eimeria* parasites with good immunogenicity have been developed as virulent or attenuated live coccidiosis vaccines that have achieved great successes in practice, especially in chickens and turkeys [6, 7]. With the rapid development of bioinformatics and the establishment of the genetic manipulation platform in apicomplexan parasites [8–11], *Eimeria* parasites have shown great potential for development as vaccine delivery vectors [12–16]. Currently, *Eimeria* vectors carrying immunoprotective or immunodominant antigen(s) of heterologous *Eimeria* species to elicit cross-protective immunities against the parental and heterologous *Eimeria* species is a novel strategy to develop next-generation coccidiosis vaccines. Using this strategy, one or several *Eimeria* species of the vaccine formulation can be removed, which can reduce the adverse pathological reaction caused by vaccination. Thus, the cost will be reduced and the safety will be improved for the novel coccidiosis vaccines [12, 16].

In our previous study, immune mapped protein 1 of *E. maxima* (EmIMP1) expressed by recombinant *E. tenella* was shown to be recognized by the host immune system and triggered a moderate EmIMP1-specific immune response [12]. Vaccination with this recombinant *E. tenella* line provided solid protection against *E. tenella* infection and partial protection against *E. maxima* infection in chickens [12]. These results indicate that *Eimeria* may be an effective antigen(s) delivery system among species with similar modes of infection and immunity. It is essential to explore strategies to improve the protection elicited by recombinant *Eimeria* against infections with heterologous species.

We hypothesized that the protective immunity could be strengthened by increasing the number of recombinant *Eimeria* lines expressing heterologous pathogens’ immunodominant antigens. To investigate this hypothesis, we constructed another recombinant *E. tenella* line expressing the recognized immunoprotective antigen, apical membrane antigen 1 of *E. maxima* (EmAMA1) [16], and then measured its immunogenicity in this study. Moreover, we assessed the improvement of the protection of chickens after co-immunization with double recombinant *Eimeria* lines, i.e. a recombinant *Eimeria* line expressing EmAMA1 (Et-EmAMA1) constructed in this study and Et-EmIMP1 [12].

## Methods

### Parasites and animals

*Eimeria tenella* (XJ strain), *E. maxima* (BJ strain) and recombinant *E. tenella* expressing EmIMP1 (Et-EmIMP1) [12] were used in this project and maintained by propagating these parasites in coccidian-free, 2–5-week-old Arbor Acres (AA) broilers, which were purchased from Beijing Arbor Acres Poultry Breeding Co., Ltd. (Beijing, China). The procedures for collection, purification and sporulation were carried out as previously described [17].

One-week- or three-week-old specific pathogen-free (SPF) chickens (White Leghorn) were purchased from Merial Animal Health Co., Ltd. (Beijing, China) and were fed a pathogen-free diet and water *ad libitum*.

### Recombinant *Eimeria* construction

**Fig. 1:**
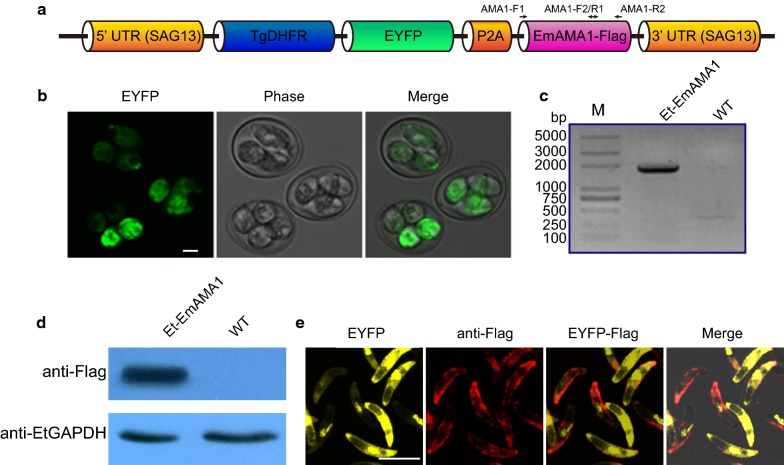
Construction of a recombinant *E. tenella* line expressing EmAMA1. **a** EmAMA1 with a flag tag was co-expressed with TgDHFR-EYFP and linked by P2A in the single expression cassette. **b** Stably transfected parasite expressing the reporter EYFP in its sporulated stage as observed by fluorescence microscopy. **c** The EmAMA1 gene was inserted into the genome of the transgenic parasites identified by PCR using AMA1-F1/AMA1-R2 primers, and the predicated PCR product was 1632 bp. **d** EmAMA1 expression in recombinant *Eimeria* was identified by Western blotting. The predicated size of EmAMA1 and the flag tag was 60.5 kDa. The mouse anti-EtGAPDH polyclonal antibody served as a loading control. **e** The exogenous EmAMA1 was primarily expressed on the cell surface and the apex of the transgenic sporozoites. The IFA experiment was conducted using the mouse anti-flag tag monoclonal antibody and the Cy3-conjugated goat anti-mouse IgG as the primary and secondary antibodies, respectively. *Scale-bars*: **b**, **e**, 5 μm

*Eimeria maxima* sporozoite cDNA was synthesized as previously described [18]. Two pairs of primers were designed to mutate the *Nde* I restriction enzyme sites (CATATG mutated to CACATG) present in the open reading frame (ORF) of EmAMA1 (GenBank: XM_013478884.1). The whole ORF (1632 bp) was amplified with overlapping PCR using the primers AMA1-F1 (5′-CAT ATG ATG TGT GGA TTG CGC GCT GC-3′)/AMA1-R2 (5′-ACC GGT GTA ATC TTG GTC AAC TAA CAC G-3′) from two fragments (1038 and 622 bp) that were amplified from the cDNA of *E. maxima* using the primers AMA1-F1/AMA1-R1 (5′-GGG GAA AAA ATA TCC ATG TGT TCC GAA G-3′) and AMA1-F2 (5′-CTT CGG AAC ACA TGG ATA TTT TTT CCC C-3′)/AMA1-R2, respectively (Fig. 1a). IMP1 in pSDEP2AIMP1S [12] was then replaced by *Nde* I and *Age* I restriction sites and AMA1 to generate pSDEP2AAMA1S. The transfection vector was linearized with *Avr* II restriction enzyme for further use.

A restriction enzyme-mediated integration (REMI) was adopted for the transfection of *E. tenella* sporozoites as previously described [19, 20]. Recombinant *E. tenella* line, Et-EmAMA1, was selected by fluorescence-activated cell sorting combined with drug pressure (Table 1). The feed was mixed with pyrimethamine (CAS:58-4-0; J&K Scientific Ltd., Beijing, China) by successive serial dilutions, and the final concentration was 150 mg/kg feed. After several generations of selection, PCR was conducted using the AMA1-F1 and AMA1-R2 primers from genomic DNA of recombinant *Eimeria* to confirm the insertion of the exogenous EmAMA1 gene. Western blot and indirect immunofluorescent assay (IFA) were conducted to confirm foreign antigen expression and distribution in recombinant parasites based on the previously described protocols, respectively [18, 21]. Recombinant *Eimeria* parasite soluble antigens were resolved by SDS-PAGE and immunoblot analysis following standard protocols with mouse anti-flag monoclonal antibody and HRP-conjugated goat anti-mouse IgG (Proteintech, Chicago, IL, USA) as primary and secondary antibodies. Poly-antibodies against *E. tenella* GAPDH served as the loading control. Indirect immunofluorescent assays were conducted to detect the distribution of EmAMA1 in the sporozoite stage of the recombinant *Eimeria* by mouse anti-flag monoclonal antibody and Cy3-conjugated goat anti-mouse IgG (Proteintech).

**Table 1:** Et-EmAMA1 selection based on EYFP expression

Generations	% EYFP expression	Selection strategy
1	32.3	Drug
2	78.3	Drug + FACS^a^
3	93.2	Drug + FACS
4	96.5	Drug^b^
5	94.7	Drug
6	94.2	–^c^

^a^Facilitating recombinant *Eimeria* parasite selection by pyrimethamine combined with fluorescence-activated cell sorting (FACS)

^b^Stabilization of the recombinant *Eimeria* population by pyrimethamine

^c^The recombinant *Eimeria* population was stable without selection pressure

### ELISA and ELISPOT

Five groups of three-week-old SPF chickens (6 chickens/group) were either left naïve (Ctrl) or were immunized by infection with 200 sporulated wild-type *E. tenella* (WT), Et-EmIMP1, Et-EmAMA1 and an equivalent mixture of Et-EmIMP1 and Et-EmAMA1 oocysts. Secondary immunization was administered at 2-week intervals with 5000 oocysts as the immunization dosage for each bird. The *E. maxima* oocysts antigen (EmAg)-specific humoral and cellular immune responses were analyzed by ELISA and enzyme-linked immunospot assay (ELISPOT), respectively. EmAg was obtained from purified sporulated oocysts as previously described [22]. EmAg (5 μg/ml) was coated onto the individual wells of the plate followed by a reaction with the serum (diluted in 1:100) collected from the birds at 2 weeks after primary and secondary (booster) immunization. The HRP-conjugated goat anti-chicken IgY Fc fragment (diluted in 1:5000; Bethyl Laboratories, Inc., Montgomery, TX, USA) was used as the secondary antibody. The optical density was measured by a microplate reader (Model 60; Bio-Rad, Hercules, CA, USA) at 450 nm.

*Eimeria maxima* parasite antigen-specific cellular immune responses revealed by IFN-γ secreting cells present in peripheral blood mononuclear cells (PBMCs) were evaluated byELISPOT at 2 weeks after secondary immunization following the established protocols [21, 23]. Briefly, 1 × 10^6^ PBMCs from the 3 randomly selected birds of each group were separately stimulated with 10 μl of PBS, 10 μl of *E. maxima* oocysts antigen (EmAg, 10 μg/ml) or 10 μl Phorbol-12-myristate-13-acetate (PMA) plus ionomycin (10 ng/ml PMA plus 5 μg/ml ionomycin). IFN-γ-secreting lymphocytes were detected after 24 h of stimulation.

### Vaccination and challenge infection

Groups of inbred SPF chickens (18 chickens/group) were either left naïve (Ctrl) or were immunized by infection with 200 sporulated wild-type *E. tenella* (WT) oocysts, 200 sporulated Et-EmIMP1 oocysts, 200 sporulated Et-EmAMA1 oocysts and 100 sporulated Et-EmIMP1 together with 100 sporulated Et-EmAMA1 oocysts at one week of age. New chopped straw litter was spread over the bottom of the cages to a depth of 5 cm. The chickens were housed under the same temperature and humidity conditions and fed a coccidian-free diet and water *ad libitum*. Six chickens from each group were separately removed to new cages (which contained a metal mesh which separated the chickens from feces) at 14, 21 and 42 dpi. The chickens were orally challenged with *E. maxima* (50 oocysts/bird) after each removal. The fecal samples were collected every day from day 5 to 12 after each challenge infection. The total number of oocysts in the feces was evaluated using a McMaster egg counting chamber after each challenge infection.

### Statistical analysis

GraphPad Prism v.6.01 (GraphPad Software) was used for statistical analysis. Differences in experimental treatments were tested using Duncan’s Multiple Range Test following ANOVA with significance reported at *P *≤ 0.05.

## Results

### Construction of a recombinant *E. tenella* line expressing *E. maxima* AMA1 (Et-EmAMA1)

In the last decade, a technical platform for the construction and selection of recombinant *Eimeria* parasites has been successfully developed [9, 20]. In this study, the transfection plasmid, pSDEP2AAMA1S (Fig. 1a) was constructed based on the previously constructed pSDEP2AIMP1S plasmid [12]. This new plasmid contains a single expression cassette in which the selected marker gene and EmAMA1 gene tagged with the flag epitope were linked by a P2A sequence, which mediates the cleavage of the dual flanking proteins [24]. The expression cassette was controlled by the EtSAG13 (*E. tenella* surface antigen 13) promoter (Fig. 1a) [25]. *Eimeria tenella* sporozoites were transfected with the linearized pSDEP2AAMA1S plasmid and then inoculated into the cloacal opening of chicks. The percentage of reporter positive recombinant *Eimeria* (Fig. 1b) in the 1st generation progeny was 32.3% (Table 1). The positive population of recombinant *Eimeria* tended to be stable after 3 generations under selection pressure (Table 1). In addition, the recombinant population remained stable without selection pressure (Table 1).

Next, we conducted serial experiments to identify the expression of exogenous EmAMA1 in the recombinant parasites. We confirmed the insertion of the EmAMA1 gene into the recombinant *Eimeria* genome by PCR using specific primers (Fig. 1c). The expression of EmAMA1 in the sporulated oocyst stage was demonstrated by Western botting using flag-tag specific-antibodies (Fig. 1d). We found that exogenous EmAMA1 was mainly expressed on the cell surface and the apex of the recombinant *Eimeria* sporozoites (Fig. 1e). These data showed that we obtained a recombinant *Eimeria* population stably expressing exogenous EmAMA1.

### Et-EmAMA1 elicited *E. maxima* antigen specific cellular immunity

**Fig. 2:**
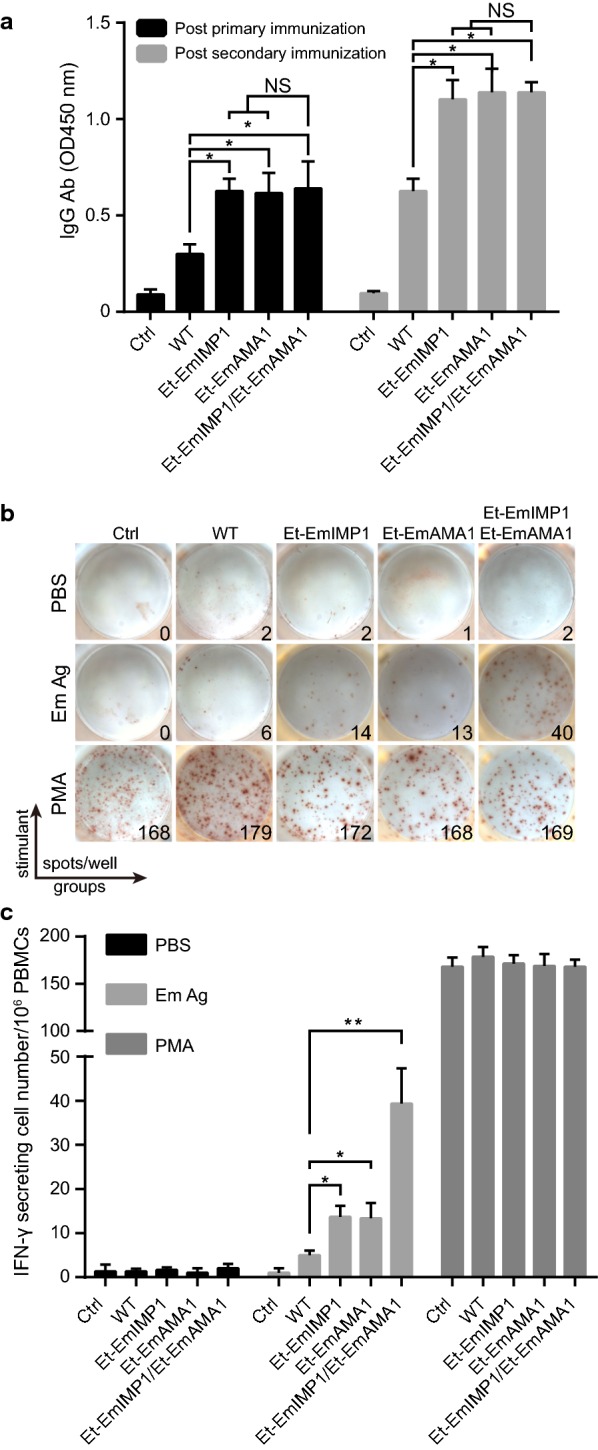
Immunization with Et-EmAMA1 and/or Et-EmIMP1 elicited *E. maxima*-specific humoral and cell-mediated immunity. **a** The EmAg-specific antibody production after primary and secondary immunization with recombinant *Eimeria* line(s) or wild-type parasites. **b**, **c** The EmAg-specific cellular immune responses after secondary immunization was analyzed by ELISPOT. The representative (**b**) and mean number (**c**) of IFN-γ-secreting lymphocytes (spots) in PBMCs from naïve (Ctrl), wild-type *E. tenella* (WT), Et-EmAMA1 only, Et-EmIMP1 only, and Et-EmAMA1 and Et-EmIMP1 immunized birds after stimulation with PBS, *E. maxima* oocyst antigens (Em Ag) and PMA plus ionomycin (PMA+Ion), (*n* = 3). *P < 0.05, **P < 0.01. *Abbreviation*: NS, no significant difference

In our previous study, we demonstrated that exogenous antigen, i.e. EmIMP1, expressed by *Eimeria* efficiently elicited EmIMP1 and *E. maxima* antigen-specific immune responses [12]. In this study, we detected the *E. maxima* antigen (EmAg)-specific immune responses elicited by Et-EmAMA1 and then focused on detecting whether immunity was enhanced by co-immunization with Et-EmAMA1 and Et-EmIMP1.We analyzed the EmAg-specific antibody production in the recombinant *Eimeria*-immunized birds and found that EmAg-specific antibody production was not significantly increased after co-immunization with Et-EmAMA1 and Et-EmIMP1 compared with immunization with Et-EmAMA1 or Et-EmIMP1 alone (Fig. 2a). We detected EmAg-specific cellular immunity after booster immunization with the single or double recombinant *Eimeria* lines, as revealed by measuring the number of IFN-γ-secreting lymphocytes in PBMCs (Fig. 2b, c). The EmAg-specific cellular immunity was shown to be significantly improved after the co-immunization with Et-EmAMA1 and Et-EmIMP1 compared to the immunization with Et-EmAMA1 or Et-EmIMP1 alone (Fig. 2b, c). The enhanced immune responses elicited by co-immunization with the double recombinant *Eimeria* lines suggest that the protection against *E. maxima* is much better than immunization with the single recombinant *Eimeria* line.

### Co-immunization with Et-EmAMA1 and Et-EmIMP1 enhanced the protective efficacy against *E. maxima* infection

**Fig. 3:**
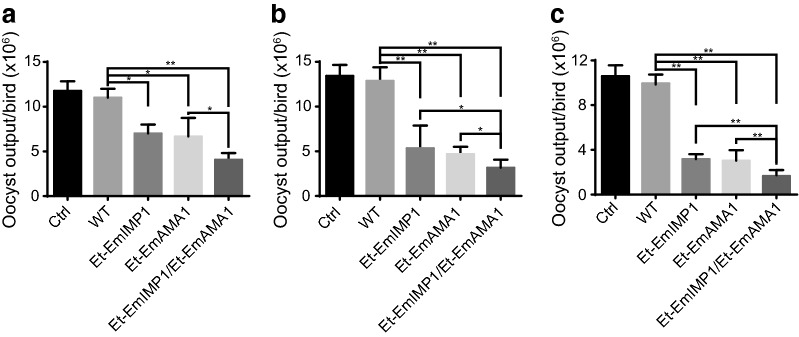
Protection against *E. maxima* infection provided by co-immunization with Et-EmAMA1 and Et-EmIMP1. **a**–**c** Oocyst output after challenge with *E. maxima* in the chickens immunized with or without Et-EmIMP1 and/or Et-EmAMA1 or its wild-type at 14 (**a**), 21 (**b**) and 42 dpi (**c**). *P < 0.05, **P < 0.01

To test whether the protective efficacy against *E. maxima* infection by immunization with double recombinant *Eimeria* lines is higher than that by immunization with a single recombinant *Eimeria* line, the birds were challenged with a low dose of *E. maxima* oocysts at different time points. We found that the oocyst output after challenge infection was significantly reduced in birds immunized with either Et-EmAMA1 or Et-EmIMP1 compared with the wild-type-immunized birds (Fig. 3). We also found that the birds immunized with Et-EmAMA1 and Et-EmIMP1 produced nearly half the number of oocysts compared to Et-EmAMA1- or Et-EmIMP1-immunized birds after challenge with *E. maxima* at 14 dpi (Fig. 3a), 21 dpi (Fig. 3b) or 42 dpi (Fig. 3c). These results demonstrated that the protective efficacy was much higher with the double recombinant *Eimeria* lines than the same dosage immunization with a single recombinant *Eimeria* line (Fig. 3). In addition, the protection against *E. maxima* infection was established as early as 14 dpi (Fig. 3a). The number of the oocyst was gradually decreased after challenge infection at 14 dpi (Fig. 3a), 21 dpi (Fig. 3b) and 42 dpi (Fig. 3c). The above results demonstrate that recombinant *Eimeria* triggered a heterologous pathogen-specific immunity and partly protected chickens against low-dose heterologous pathogen infection.

## Discussion

In this study, we successfully obtained a recombinant *E. tenella* line expressing heterologous EmAMA1 and tested its immunogenicity. We found that both Et-EmAMA1 and Et-EmIMP1 [12] could protect chickens against heterologous *E. maxima* infection, and the protection could be enhanced by co-immunization with double recombinant *Eimeria* lines. In addition to previous findings, these results suggested that *Eimeria* is an effective vaccine vector that can carry and deliver *Eimeria* and other pathogens’ antigens to the host immune system and trigger specific immune responses [12–15]. Our results also suggest that increasing the number of recombinant *Eimeria* lines may be an effective approach to strengthen the protective immunity against infections with heterologous pathogens. This is of great significance for developing next-generation coccidiosis vaccines that can simplify formulations of recombinant vaccines for animals and humans.

Our results show that recombinant *Eimeria* efficiently elicited heterologous antigen specific antibody production and cell-mediated immunity. The cell-mediated immunity rather than antibody production was improved when co-immunized with double recombinant *Eimeria* lines (Fig. 2) suggest that the enhanced protection by co-immunization is related to cellular immunity. Parallel experiments demonstrated that the secretion or cell surface display of EmAMA1 in recombinant *E. tenella* elicited EmAMA1 specific-antibody production. Multiple immunizations with recombinant *Eimeria* could partly protect the chickens against a moderate dose (300 oocysts/bird) of *E. maxima* infection [16]. The reduced oocyst production after challenge infection in recombinant *Eimeria* immunized birds may be related to the reduced serum IL-10 level [16]. Using EmIMP1 in recombinant protein or EmAMA1 in DNA formulations has been reported to achieve a reduction of approximately 45% in parasite production in small-scale vaccination trials [26, 27]. The reduction in oocyst production was 30–90% using *E. tenella* or *E. acervulina* antigens in recombinant protein, DNA or live-vector formulations with or without cytokines as adjuvants [27, 28]. Moreover, the protection against *E. tenella*, *E. necatrix*, *E. maxima* or *E. acervulina* infection was also reported to be enhanced using multivalent epitope DNA vaccines from multiple antigens compared to a single antigen [29–34]. Although those subunit or recombinant vaccines achieved promising successes in the laboratory and are much safer and less expensive than live anticoccidial vaccines, no large-scale trials have been reported [27]. One explanation may be the lack of automation systems for mass immunization and that chickens experience more stress with intramuscular injection of recombinant protein and DNA vaccines than with oral immunization with live anticoccidial vaccines [35–37]. Another explanation could be that the achieved protection through recombinant protein and DNA vaccines can only be detected after one or two booster immunizations, whereas a single oral immunization with live oocysts can elicit long-term protective immunity [6, 38].

To date, no matter the form of antigen delivery, i.e. recombinant protein, DNA or live vector including *Eimeria*, antigens can elicit complete protection as effective as live anticoccidial vaccines in the field. Our present results show promise for improving immune protection by recombinant *Eimeria* by increasing the number or category of heterologous antigens. Solutions include but are not limited to: (i) using advanced biotechnological tools to discover new immunodominant antigen(s) of *Eimeria*, such as IMP1 [26]; (ii) improving the heterologous antigen expression level by using a stronger promoter or increasing the gene copy number, etc. [24]; (iii) co-expressing multiple immunodominant antigens mediated by P2A in one recombinant *Eimeria* line or by double or multiple expression cassette [24, 39]; (iv) optimizing the antigens’ location in recombinant *Eimeria* for efficient recognition by host immune system [18]; and (v) fusion expression of heterologous antigens with cytokine(s) or other molecular adjuvants [21, 25, 40].

## Conclusions

Recombinant *E. tenella* expressing an immunodominant antigen of *E. maxima* (EmAMA1) elicited partial protection against *E. maxima* infection in chickens, and the protection was improved by co-immunization with two recombinant *Eimeria* lines (Et-EmIMP1 and Et-EmAMA1). Our results provide a good basis for further development of next-generations coccidiosis vaccines and possess great significance for developing other recombinant vaccines for animals and humans.

## Data Availability

All data generated or analyzed during this study are included in this published article.

## Abbreviations

IMP1: immune mapped protein 1
AMA1: apical membrane antigen 1
SAG13: surface antigen 13
EmAg: *Eimeria maxima* antigen
PBMCs: peripheral blood mononuclear cells
dpi: days post-immunization
dpc: days post-challenge
ORF: open reading frame
REMI: restriction enzyme-mediated integration
IFA: indirect immunofluorescent assay
SPF: specific pathogen free
ELISPOT: enzyme-linked immunospot assay
PMA: phorbol-12-myristate-13-acetate
